# Perspektiven für die Haushaltspolitik der Europäischen Union

**DOI:** 10.1007/s10273-021-2844-2

**Published:** 2021-02-20

**Authors:** Überblick

**Keywords:** H12, H31, H61, H77

## Abstract

Die Europäische Union hat mit „NextGenerationEU“ (NGEU) einen Fonds aufgelegt, um die wirtschaftliche Erholung ihrer Mitgliedstaaten während und nach der Corona-Pandemie zu unterstützen. Mit dem Fonds sollen die Kohäsion sowie der grüne und digitale Wandel in Europa befördert werden. Gleichzeitig hat die EU ihre Haushaltsmittel im Rahmen des Mehrjährigen Finanzrahmens 2021 bis 2027 nur leicht erhöht. Die finanzielle Unterstützung durch die EU wird positiv bewertet. Allerdings bleibt fraglich, wo die nationalen Prioritäten liegen und ob die gesteckten Ziele tatsächlich strukturelle Veränderungen im Sinne der EU ermöglichen. In jedem Fall verändert NGEU die Finanzarchitektur der EU, da der Fonds über gemeinsame Anleihen finanziert werden soll. Welche Folgen dies für die Struktur der EU-Finanzen langfristig haben kann und welche Reformansätze sinnvoll sind, ist Gegenstand der Debatte.

## Abstract (en)

The European Union has launched the Next Generation EU (NGEU) fund to support the economic recovery of its member states during and after the coronavirus pandemic. The fund aims to promote cohesion and the green and digital transformation in Europe. At the same time, the EU has only slightly increased its budget under the Multiannual Financial Framework 2021 to 2027. The financial support from the EU is assessed positively. However, it remains questionable where the national priorities lie and whether the set goals actually enable structural changes in the EU. In any case, NGEU changes the financial architecture of the EU, as the fund is to be financed through common bonds. What consequences this may have for the structure of EU finances in the long term and which reform approaches make sense is the subject of debate.

